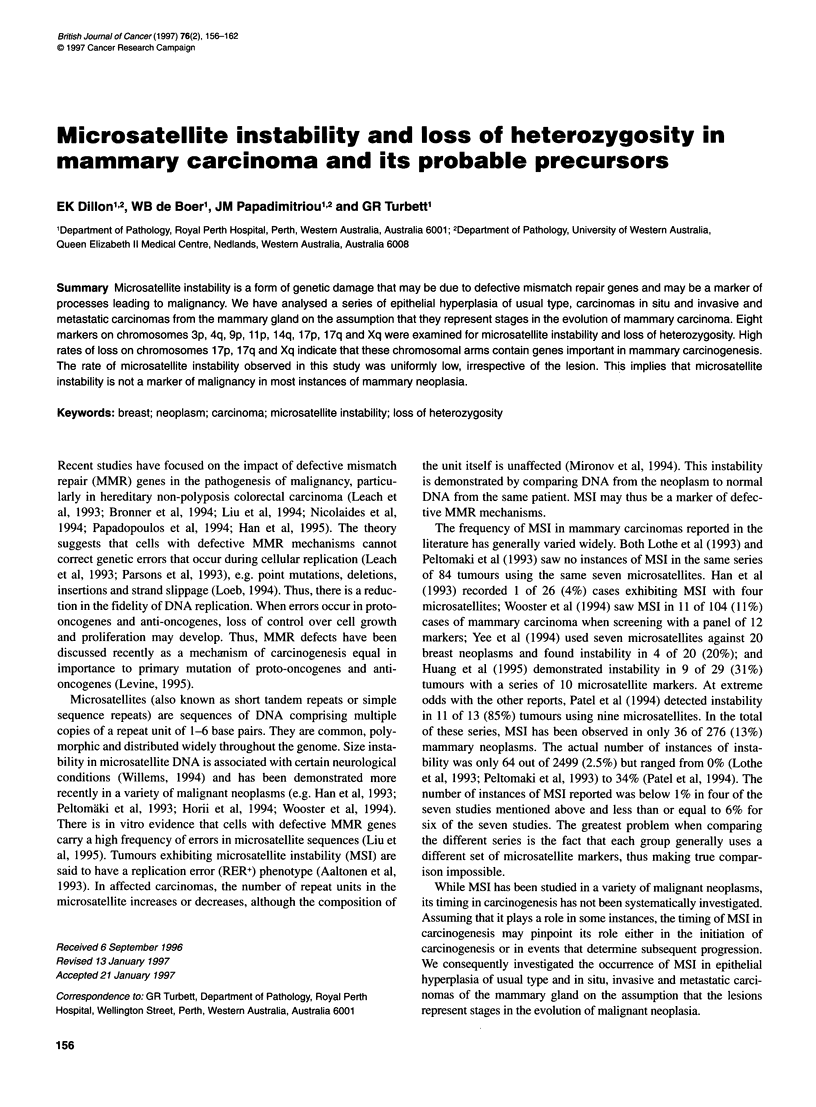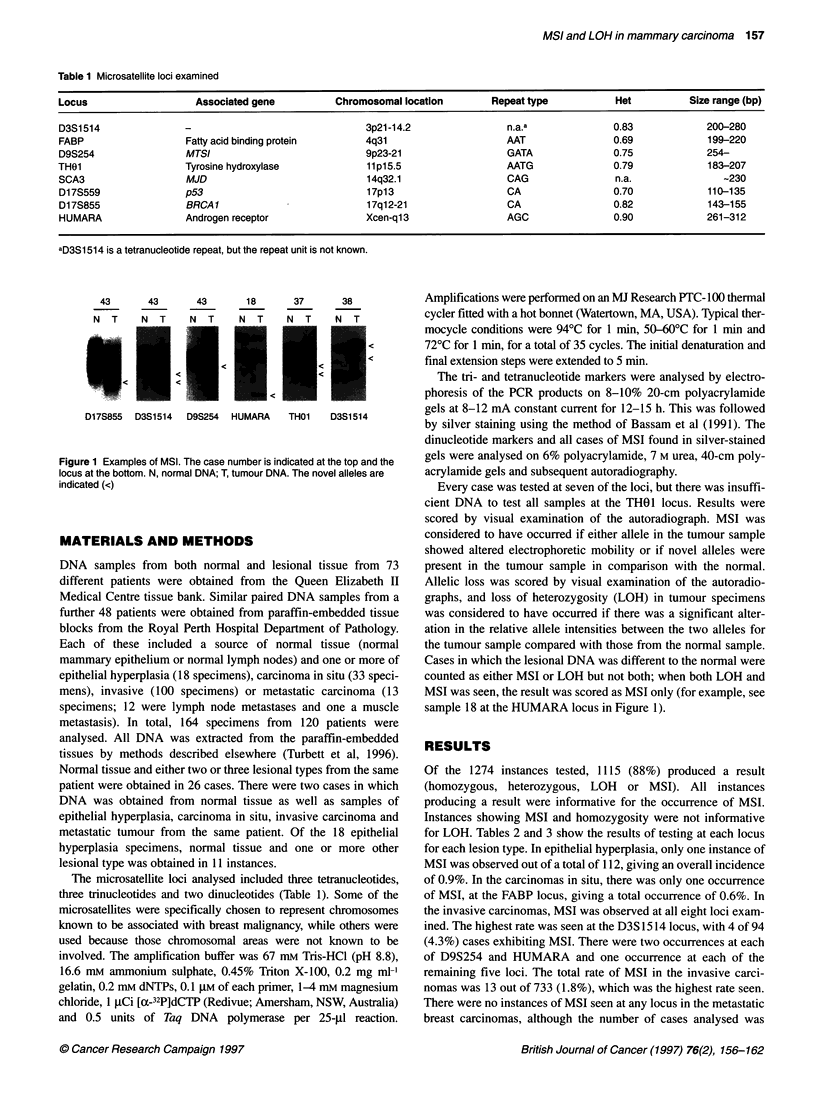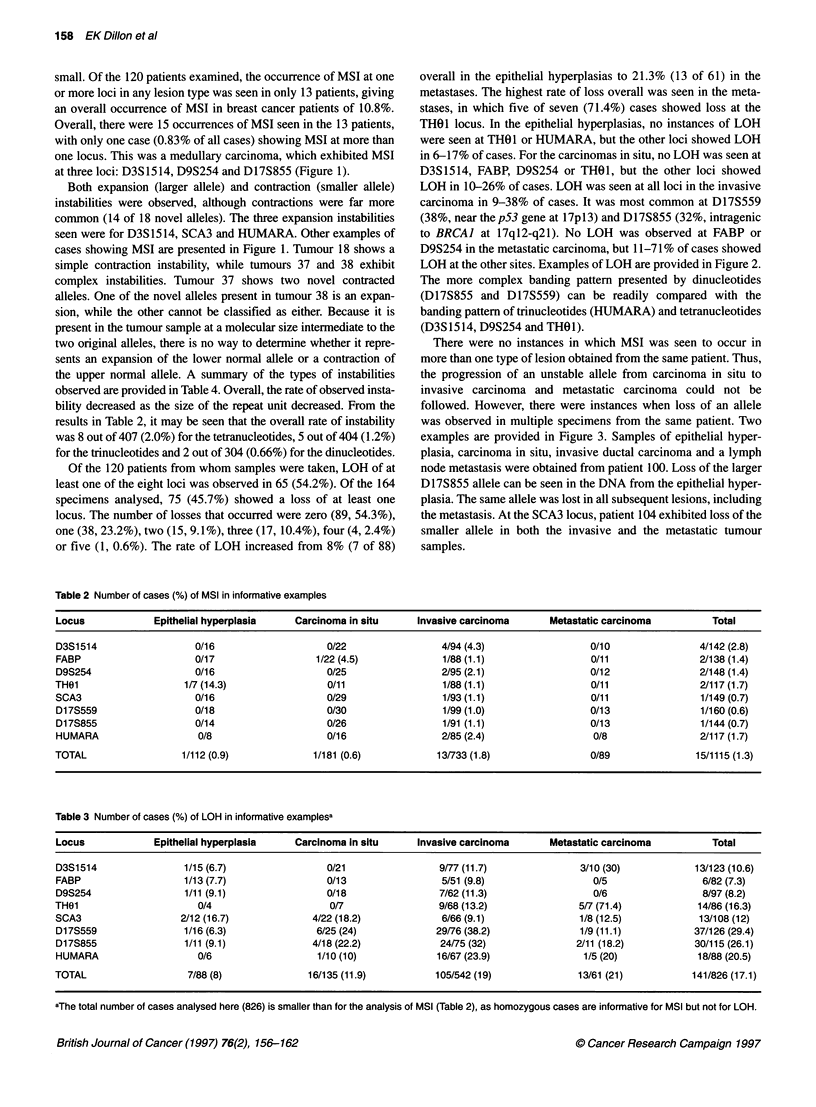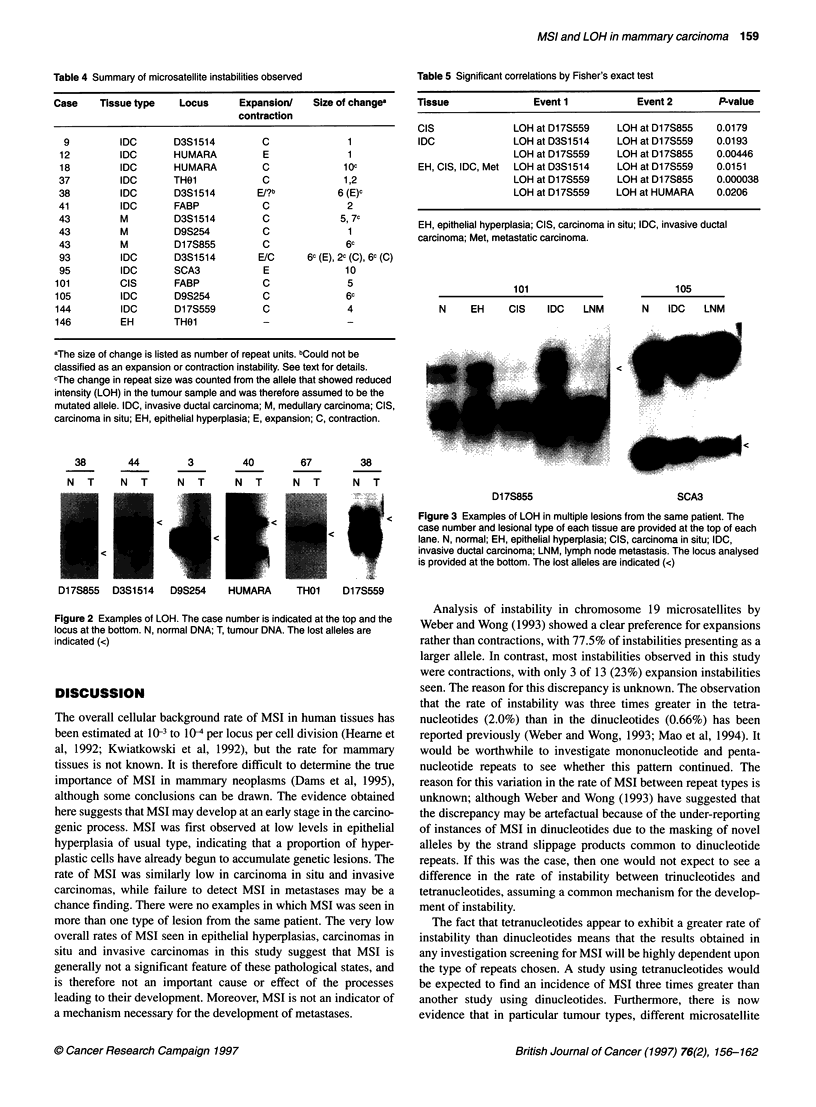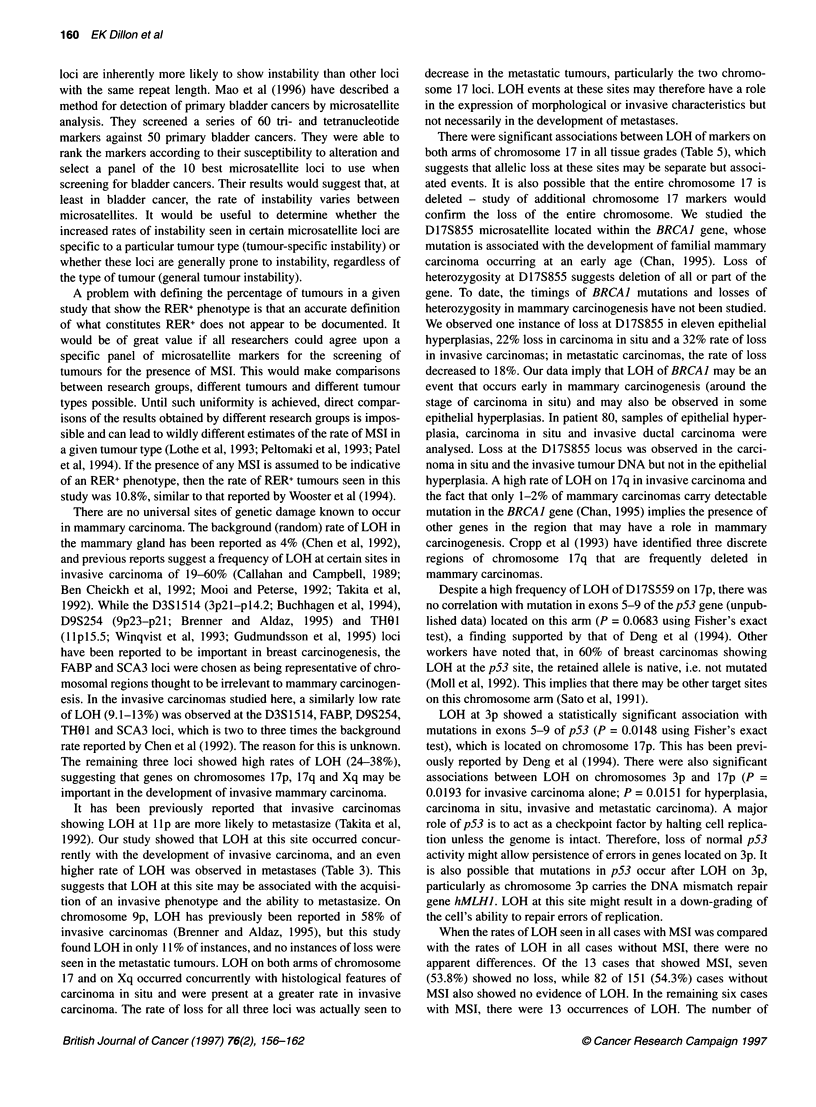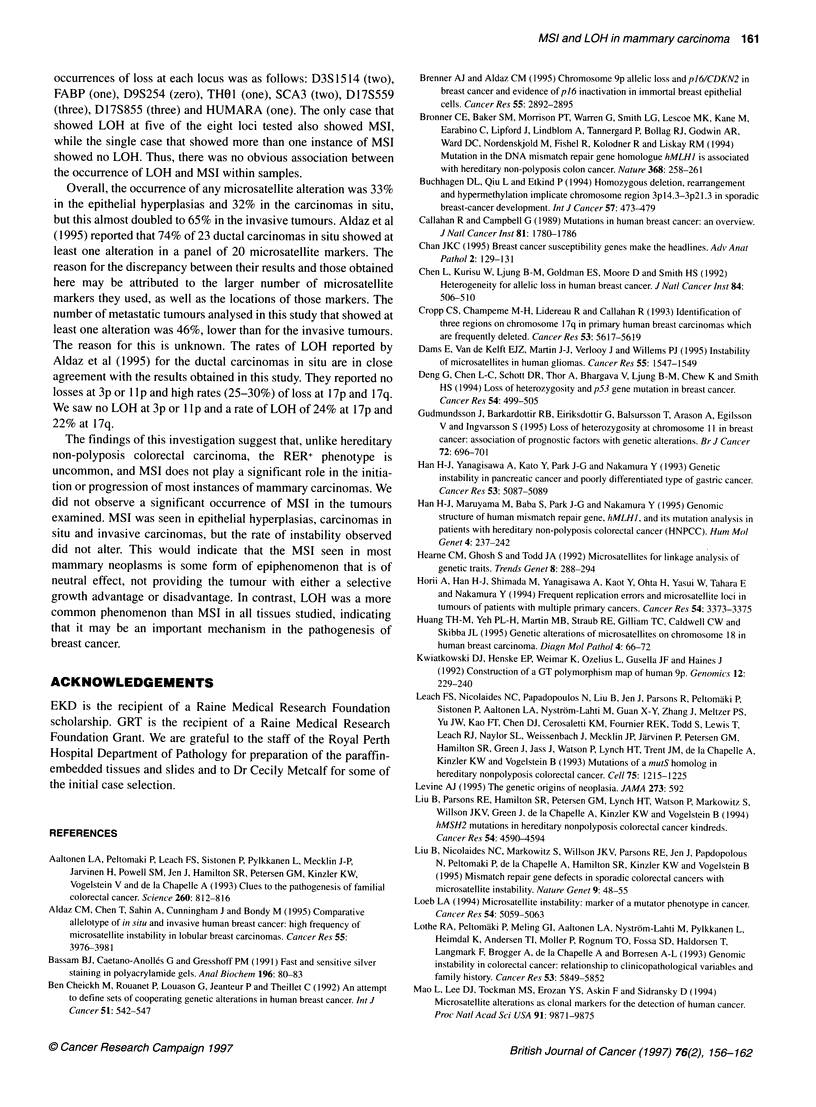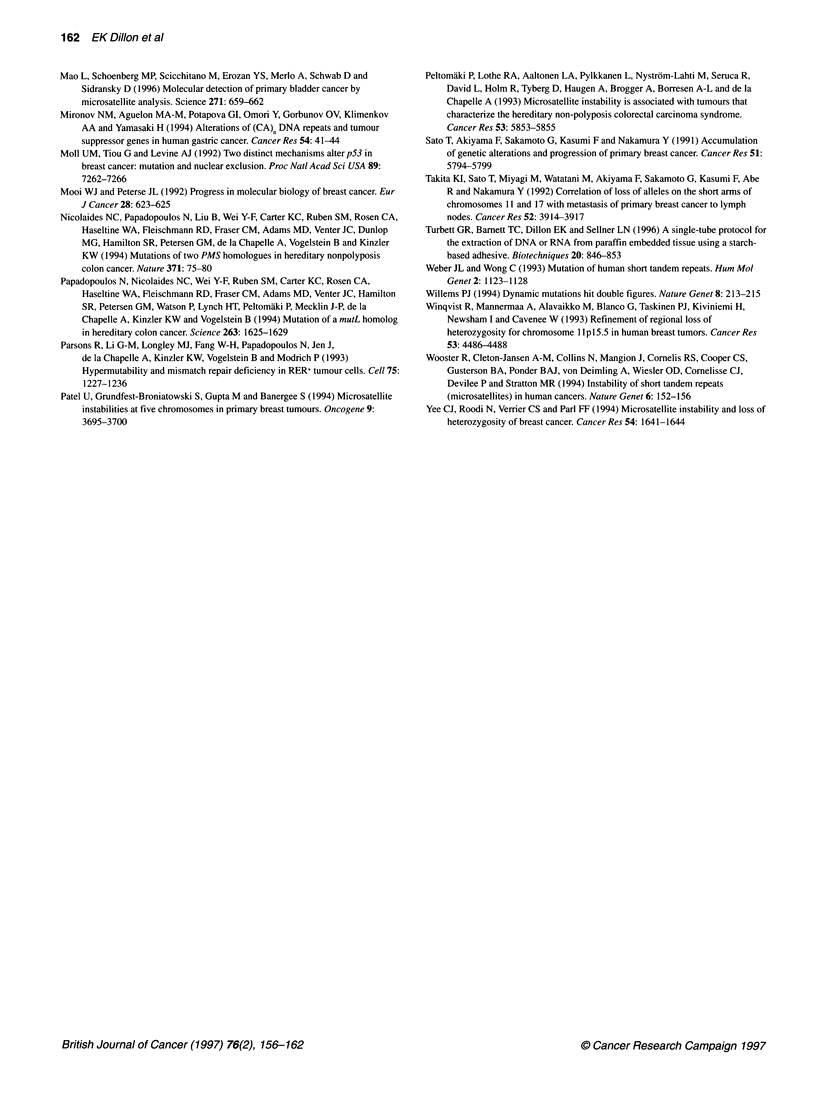# Microsatellite instability and loss of heterozygosity in mammary carcinoma and its probable precursors.

**DOI:** 10.1038/bjc.1997.357

**Published:** 1997

**Authors:** E. K. Dillon, W. B. de Boer, J. M. Papadimitriou, G. R. Turbett

**Affiliations:** Department of Pathology, Royal Perth Hospital, Western Australia.

## Abstract

**Images:**


British Journal of Cancer (1997) 76(2), 156–162
© 1997 Cancer Research Campaign

### Microsatellite instability and loss of heterozygosity in mammary carcinoma and its probable precursors

**EK Dillon[1,2], WB de Boer[1], JM Papadimitriou[1,2] and GR Turbett[1]**

[1]Department of Pathology, Royal Perth Hospital, Perth, Western Australia, Australia 6001; [2]Department of Pathology, University of Western Australia, Queen Elizabeth II Medical Centre, Nedlands, Western Australia, Australia 6008

**Summary** Microsatellite instability is a form of genetic damage that may be due to defective mismatch repair genes and may be a marker of processes leading to malignancy. We have analysed a series of epithelial hyperplasia of usual type, carcinomas in situ and invasive and metastatic carcinomas from the mammary gland on the assumption that they represent stages in the evolution of mammary carcinoma. Eight markers on chromosomes 3p, 4q, 9p, 11p, 14q, 17p, 17q and Xq were examined for microsatellite instability and loss of heterozygosity. High rates of loss on chromosomes 17p, 17q and Xq indicate that these chromosomal arms contain genes important in mammary carcinogenesis. The rate of microsatellite instability observed in this study was uniformly low, irrespective of the lesion. This implies that microsatellite instability is not a marker of malignancy in most instances of mammary neoplasia.

**Keywords:** breast; neoplasm; carcinoma; microsatellite instability; loss of heterozygosity

Recent studies have focused on the impact of defective mismatch repair (MMR) genes in the pathogenesis of malignancy, particularly in hereditary non-polyposis colorectal carcinoma (Leach et al, 1993; Bronner et al, 1994; Liu et al, 1994; Nicolaides et al, 1994; Papadopoulos et al, 1994; Han et al, 1995). The theory suggests that cells with defective MMR mechanisms cannot correct genetic errors that occur during cellular replication (Leach et al, 1993; Parsons et al, 1993), e.g. point mutations, deletions, insertions and strand slippage (Loeb, 1994). Thus, there is a reduction in the fidelity of DNA replication. When errors occur in proto-oncogenes and anti-oncogenes, loss of control over cell growth and proliferation may develop. Thus, MMR defects have been discussed recently as a mechanism of carcinogenesis equal in importance to primary mutation of proto-oncogenes and anti-oncogenes (Levine, 1995).

Microsatellites (also known as short tandem repeats or simple sequence repeats) are sequences of DNA comprising multiple copies of a repeat unit of 1–6 base pairs. They are common, polymorphic and distributed widely throughout the genome. Size instability in microsatellite DNA is associated with certain neurological conditions (Willems, 1994) and has been demonstrated more recently in a variety of malignant neoplasms (e.g. Han et al, 1993; Peltomäki et al, 1993; Horii et al, 1994; Wooster et al, 1994). There is in vitro evidence that cells with defective MMR genes carry a high frequency of errors in microsatellite sequences (Liu et al, 1995). Tumours exhibiting microsatellite instability (MSI) are said to have a replication error ($RER^+$) phenotype (Aaltonen et al, 1993). In affected carcinomas, the number of repeat units in the microsatellite increases or decreases, although the composition of the unit itself is unaffected (Mironov et al, 1994). This instability is demonstrated by comparing DNA from the neoplasm to normal DNA from the same patient. MSI may thus be a marker of defective MMR mechanisms.

The frequency of MSI in mammary carcinomas reported in the literature has generally varied widely. Both Lothe et al (1993) and Peltomaki et al (1993) saw no instances of MSI in the same series of 84 tumours using the same seven microsatellites. Han et al (1993) recorded 1 of 26 (4%) cases exhibiting MSI with four microsatellites; Wooster et al (1994) saw MSI in 11 of 104 (11%) cases of mammary carcinoma when screening with a panel of 12 markers; Yee et al (1994) used seven microsatellites against 20 breast neoplasms and found instability in 4 of 20 (20%); and Huang et al (1995) demonstrated instability in 9 of 29 (31%) tumours with a series of 10 microsatellite markers. At extreme odds with the other reports, Patel et al (1994) detected instability in 11 of 13 (85%) tumours using nine microsatellites. In the total of these series, MSI has been observed in only 36 of 276 (13%) mammary neoplasms. The actual number of instances of instability was only 64 out of 2499 (2.5%) but ranged from 0% (Lothe et al, 1993; Peltomaki et al, 1993) to 34% (Patel et al, 1994). The number of instances of MSI reported was below 1% in four of the seven studies mentioned above and less than or equal to 6% for six of the seven studies. The greatest problem when comparing the different series is the fact that each group generally uses a different set of microsatellite markers, thus making true comparison impossible.

While MSI has been studied in a variety of malignant neoplasms, its timing in carcinogenesis has not been systematically investigated. Assuming that it plays a role in some instances, the timing of MSI in carcinogenesis may pinpoint its role either in the initiation of carcinogenesis or in events that determine subsequent progression. We consequently investigated the occurrence of MSI in epithelial hyperplasia of usual type and in situ, invasive and metastatic carcinomas of the mammary gland on the assumption that the lesions represent stages in the evolution of malignant neoplasia.

*Received 6 September 1996*
*Revised 13 January 1997*
*Accepted 21 January 1997*

*Correspondence to:* GR Turbett, Department of Pathology, Royal Perth Hospital, Wellington Street, Perth, Western Australia, Australia 6001

**Table 1** Microsatellite loci examined

| Locus | Associated gene | Chromosomal location | Repeat type | Het | Size range (bp) |
|---|---|---|---|---|---|
| D3S1514 | – | 3p21-14.2 | n.a.[a] | 0.83 | 200–280 |
| FABP | Fatty acid binding protein | 4q31 | AAT | 0.69 | 199–220 |
| D9S254 | *MTSI* | 9p23-21 | GATA | 0.75 | 254– |
| THθ1 | Tyrosine hydroxylase | 11p15.5 | AATG | 0.79 | 183–207 |
| SCA3 | *MJD* | 14q32.1 | CAG | n.a. | ~230 |
| D17S559 | *p53* | 17p13 | CA | 0.70 | 110–135 |
| D17S855 | *BRCA1* | 17q12-21 | CA | 0.82 | 143–155 |
| HUMARA | Androgen receptor | Xcen-q13 | AGC | 0.90 | 261–312 |

[a]D3S1514 is a tetranucleotide repeat, but the repeat unit is not known.

**Figure 1** Examples of MSI. The case number is indicated at the top and the locus at the bottom. N, normal DNA; T, tumour DNA. The novel alleles are indicated (<)

### MATERIALS AND METHODS

DNA samples from both normal and lesional tissue from 73 different patients were obtained from the Queen Elizabeth II Medical Centre tissue bank. Similar paired DNA samples from a further 48 patients were obtained from paraffin-embedded tissue blocks from the Royal Perth Hospital Department of Pathology. Each of these included a source of normal tissue (normal mammary epithelium or normal lymph nodes) and one or more of epithelial hyperplasia (18 specimens), carcinoma in situ (33 specimens), invasive (100 specimens) or metastatic carcinoma (13 specimens; 12 were lymph node metastases and one a muscle metastasis). In total, 164 specimens from 120 patients were analysed. All DNA was extracted from the paraffin-embedded tissues by methods described elsewhere (Turbett et al, 1996). Normal tissue and either two or three lesional types from the same patient were obtained in 26 cases. There were two cases in which DNA was obtained from normal tissue as well as samples of epithelial hyperplasia, carcinoma in situ, invasive carcinoma and metastatic tumour from the same patient. Of the 18 epithelial hyperplasia specimens, normal tissue and one or more other lesional type was obtained in 11 instances.

The microsatellite loci analysed included three tetranucleotides, three trinucleotides and two dinucleotides (Table 1). Some of the microsatellites were specifically chosen to represent chromosomes known to be associated with breast malignancy, while others were used because those chromosomal areas were not known to be involved. The amplification buffer was 67 mM Tris-HCl (pH 8.8), 16.6 mM ammonium sulphate, 0.45% Triton X-100, 0.2 mg ml$^{-1}$ gelatin, 0.2 mM dNTPs, 0.1 µM of each primer, 1–4 mM magnesium chloride, 1 µCi [$\alpha$-$^{32}$P]dCTP (Redivue; Amersham, NSW, Australia) and 0.5 units of *Taq* DNA polymerase per 25-µl reaction. Amplifications were performed on an MJ Research PTC-100 thermal cycler fitted with a hot bonnet (Watertown, MA, USA). Typical thermocycle conditions were 94°C for 1 min, 50–60°C for 1 min and 72°C for 1 min, for a total of 35 cycles. The initial denaturation and final extension steps were extended to 5 min.

The tri- and tetranucleotide markers were analysed by electrophoresis of the PCR products on 8–10% 20-cm polyacrylamide gels at 8–12 mA constant current for 12–15 h. This was followed by silver staining using the method of Bassam et al (1991). The dinucleotide markers and all cases of MSI found in silver-stained gels were analysed on 6% polyacrylamide, 7 M urea, 40-cm polyacrylamide gels and subsequent autoradiography.

Every case was tested at seven of the loci, but there was insufficient DNA to test all samples at the THθ1 locus. Results were scored by visual examination of the autoradiograph. MSI was considered to have occurred if either allele in the tumour sample showed altered electrophoretic mobility or if novel alleles were present in the tumour sample in comparison with the normal. Allelic loss was scored by visual examination of the autoradiographs, and loss of heterozygosity (LOH) in tumour specimens was considered to have occurred if there was a significant alteration in the relative allele intensities between the two alleles for the tumour sample compared with those from the normal sample. Cases in which the lesional DNA was different to the normal were counted as either MSI or LOH but not both; when both LOH and MSI was seen, the result was scored as MSI only (for example, see sample 18 at the HUMARA locus in Figure 1).

### RESULTS

Of the 1274 instances tested, 1115 (88%) produced a result (homozygous, heterozygous, LOH or MSI). All instances producing a result were informative for the occurrence of MSI. Instances showing MSI and homozygosity were not informative for LOH. Tables 2 and 3 show the results of testing at each locus for each lesion type. In epithelial hyperplasia, only one instance of MSI was observed out of a total of 112, giving an overall incidence of 0.9%. In the carcinomas in situ, there was only one occurrence of MSI, at the FABP locus, giving a total occurrence of 0.6%. In the invasive carcinomas, MSI was observed at all eight loci examined. The highest rate was seen at the D3S1514 locus, with 4 of 94 (4.3%) cases exhibiting MSI. There were two occurrences at each of D9S254 and HUMARA and one occurrence at each of the remaining five loci. The total rate of MSI in the invasive carcinomas was 13 out of 733 (1.8%), which was the highest rate seen. There were no instances of MSI seen at any locus in the metastatic breast carcinomas, although the number of cases analysed was

© Cancer Research Campaign 1997

small. Of the 120 patients examined, the occurrence of MSI at one or more loci in any lesion type was seen in only 13 patients, giving an overall occurrence of MSI in breast cancer patients of 10.8%. Overall, there were 15 occurrences of MSI seen in the 13 patients, with only one case (0.83% of all cases) showing MSI at more than one locus. This was a medullary carcinoma, which exhibited MSI at three loci: D3S1514, D9S254 and D17S855 (Figure 1).

Both expansion (larger allele) and contraction (smaller allele) instabilities were observed, although contractions were far more common (14 of 18 novel alleles). The three expansion instabilities seen were for D3S1514, SCA3 and HUMARA. Other examples of cases showing MSI are presented in Figure 1. Tumour 18 shows a simple contraction instability, while tumours 37 and 38 exhibit complex instabilities. Tumour 37 shows two novel contracted alleles. One of the novel alleles present in tumour 38 is an expansion, while the other cannot be classified as either. Because it is present in the tumour sample at a molecular size intermediate to the two original alleles, there is no way to determine whether it represents an expansion of the lower normal allele or a contraction of the upper normal allele. A summary of the types of instabilities observed are provided in Table 4. Overall, the rate of observed instability decreased as the size of the repeat unit decreased. From the results in Table 2, it may be seen that the overall rate of instability was 8 out of 407 (2.0%) for the tetranucleotides, 5 out of 404 (1.2%) for the trinucleotides and 2 out of 304 (0.66%) for the dinucleotides.

Of the 120 patients from whom samples were taken, LOH of at least one of the eight loci was observed in 65 (54.2%). Of the 164 specimens analysed, 75 (45.7%) showed a loss of at least one locus. The number of losses that occurred were zero (89, 54.3%), one (38, 23.2%), two (15, 9.1%), three (17, 10.4%), four (4, 2.4%) or five (1, 0.6%). The rate of LOH increased from 8% (7 of 88) overall in the epithelial hyperplasias to 21.3% (13 of 61) in the metastases. The highest rate of loss overall was seen in the metastases, in which five of seven (71.4%) cases showed loss at the THθ1 locus. In the epithelial hyperplasias, no instances of LOH were seen at THθ1 or HUMARA, but the other loci showed LOH in 6–17% of cases. For the carcinomas in situ, no LOH was seen at D3S1514, FABP, D9S254 or THθ1, but the other loci showed LOH in 10–26% of cases. LOH was seen at all loci in the invasive carcinoma in 9–38% of cases. It was most common at D17S559 (38%, near the *p53* gene at 17p13) and D17S855 (32%, intragenic to *BRCA1* at 17q12-q21). No LOH was observed at FABP or D9S254 in the metastatic carcinoma, but 11–71% of cases showed LOH at the other sites. Examples of LOH are provided in Figure 2. The more complex banding pattern presented by dinucleotides (D17S855 and D17S559) can be readily compared with the banding pattern of trinucleotides (HUMARA) and tetranucleotides (D3S1514, D9S254 and THθ1).

There were no instances in which MSI was seen to occur in more than one type of lesion obtained from the same patient. Thus, the progression of an unstable allele from carcinoma in situ to invasive carcinoma and metastatic carcinoma could not be followed. However, there were instances when loss of an allele was observed in multiple specimens from the same patient. Two examples are provided in Figure 3. Samples of epithelial hyperplasia, carcinoma in situ, invasive ductal carcinoma and a lymph node metastasis were obtained from patient 100. Loss of the larger D17S855 allele can be seen in the DNA from the epithelial hyperplasia. The same allele was lost in all subsequent lesions, including the metastasis. At the SCA3 locus, patient 104 exhibited loss of the smaller allele in both the invasive and the metastatic tumour samples.

**Table 2** Number of cases (%) of MSI in informative examples

| Locus | Epithelial hyperplasia | Carcinoma in situ | Invasive carcinoma | Metastatic carcinoma | Total |
|---|---|---|---|---|---|
| D3S1514 | 0/16 | 0/22 | 4/94 (4.3) | 0/10 | 4/142 (2.8) |
| FABP | 0/17 | 1/22 (4.5) | 1/88 (1.1) | 0/11 | 2/138 (1.4) |
| D9S254 | 0/16 | 0/25 | 2/95 (2.1) | 0/12 | 2/148 (1.4) |
| THθ1 | 1/7 (14.3) | 0/11 | 1/88 (1.1) | 0/11 | 2/117 (1.7) |
| SCA3 | 0/16 | 0/29 | 1/93 (1.1) | 0/11 | 1/149 (0.7) |
| D17S559 | 0/18 | 0/30 | 1/99 (1.0) | 0/13 | 1/160 (0.6) |
| D17S855 | 0/14 | 0/26 | 1/91 (1.1) | 0/13 | 1/144 (0.7) |
| HUMARA | 0/8 | 0/16 | 2/85 (2.4) | 0/8 | 2/117 (1.7) |
| TOTAL | 1/112 (0.9) | 1/181 (0.6) | 13/733 (1.8) | 0/89 | 15/1115 (1.3) |

**Table 3** Number of cases (%) of LOH in informative examples[a]

| Locus | Epithelial hyperplasia | Carcinoma in situ | Invasive carcinoma | Metastatic carcinoma | Total |
|---|---|---|---|---|---|
| D3S1514 | 1/15 (6.7) | 0/21 | 9/77 (11.7) | 3/10 (30) | 13/123 (10.6) |
| FABP | 1/13 (7.7) | 0/13 | 5/51 (9.8) | 0/5 | 6/82 (7.3) |
| D9S254 | 1/11 (9.1) | 0/18 | 7/62 (11.3) | 0/6 | 8/97 (8.2) |
| THθ1 | 0/4 | 0/7 | 9/68 (13.2) | 5/7 (71.4) | 14/86 (16.3) |
| SCA3 | 2/12 (16.7) | 4/22 (18.2) | 6/66 (9.1) | 1/8 (12.5) | 13/108 (12) |
| D17S559 | 1/16 (6.3) | 6/25 (24) | 29/76 (38.2) | 1/9 (11.1) | 37/126 (29.4) |
| D17S855 | 1/11 (9.1) | 4/18 (22.2) | 24/75 (32) | 2/11 (18.2) | 30/115 (26.1) |
| HUMARA | 0/6 | 1/10 (10) | 16/67 (23.9) | 1/5 (20) | 18/88 (20.5) |
| TOTAL | 7/88 (8) | 16/135 (11.9) | 105/542 (19) | 13/61 (21) | 141/826 (17.1) |

[a]The total number of cases analysed here (826) is smaller than for the analysis of MSI (Table 2), as homozygous cases are informative for MSI but not for LOH.

 © Cancer Research Campaign 1997

**Table 4** Summary of microsatellite instabilities observed

| Case | Tissue type | Locus | Expansion/ contraction | Size of change[a] |
|---|---|---|---|---|
| 9 | IDC | D3S1514 | C | 1 |
| 12 | IDC | HUMARA | E | 1 |
| 18 | IDC | HUMARA | C | 10[c] |
| 37 | IDC | THθ1 | C | 1,2 |
| 38 | IDC | D3S1514 | E/?[b] | 6 (E)[c] |
| 41 | IDC | FABP | C | 2 |
| 43 | M | D3S1514 | C | 5, 7[c] |
| 43 | M | D9S254 | C | 1 |
| 43 | M | D17S855 | C | 6[c] |
| 93 | IDC | D3S1514 | E/C | 6[c] (E), 2[c] (C), 6[c] (C) |
| 95 | IDC | SCA3 | E | 10 |
| 101 | CIS | FABP | C | 5 |
| 105 | IDC | D9S254 | C | 6[c] |
| 144 | IDC | D17S559 | C | 4 |
| 146 | EH | THθ1 | – | – |

[a]The size of change is listed as number of repeat units. [b]Could not be classified as an expansion or contraction instability. See text for details. [c]The change in repeat size was counted from the allele that showed reduced intensity (LOH) in the tumour sample and was therefore assumed to be the mutated allele. IDC, invasive ductal carcinoma; M, medullary carcinoma; CIS, carcinoma in situ; EH, epithelial hyperplasia; E, expansion; C, contraction.

**Figure 2** Examples of LOH. The case number is indicated at the top and the locus at the bottom. N, normal DNA; T, tumour DNA. The lost alleles are indicated (<)

### DISCUSSION

The overall cellular background rate of MSI in human tissues has been estimated at $10^{-3}$ to $10^{-4}$ per locus per cell division (Hearne et al, 1992; Kwiatkowski et al, 1992), but the rate for mammary tissues is not known. It is therefore difficult to determine the true importance of MSI in mammary neoplasms (Dams et al, 1995), although some conclusions can be drawn. The evidence obtained here suggests that MSI may develop at an early stage in the carcinogenic process. MSI was first observed at low levels in epithelial hyperplasia of usual type, indicating that a proportion of hyperplastic cells have already begun to accumulate genetic lesions. The rate of MSI was similarly low in carcinoma in situ and invasive carcinomas, while failure to detect MSI in metastases may be a chance finding. There were no examples in which MSI was seen in more than one type of lesion from the same patient. The very low overall rates of MSI seen in epithelial hyperplasias, carcinomas in situ and invasive carcinomas in this study suggest that MSI is generally not a significant feature of these pathological states, and is therefore not an important cause or effect of the processes leading to their development. Moreover, MSI is not an indicator of a mechanism necessary for the development of metastases.

**Table 5** Significant correlations by Fisher's exact test

| Tissue | Event 1 | Event 2 | *P*-value |
|---|---|---|---|
| CIS | LOH at D17S559 | LOH at D17S855 | 0.0179 |
| IDC | LOH at D3S1514 | LOH at D17S559 | 0.0193 |
| | LOH at D17S559 | LOH at D17S855 | 0.00446 |
| EH, CIS, IDC, Met | LOH at D3S1514 | LOH at D17S559 | 0.0151 |
| | LOH at D17S559 | LOH at D17S855 | 0.000038 |
| | LOH at D17S559 | LOH at HUMARA | 0.0206 |

EH, epithelial hyperplasia; CIS, carcinoma in situ; IDC, invasive ductal carcinoma; Met, metastatic carcinoma.

**Figure 3** Examples of LOH in multiple lesions from the same patient. The case number and lesional type of each tissue are provided at the top of each lane. N, normal; EH, epithelial hyperplasia; CIS, carcinoma in situ; IDC, invasive ductal carcinoma; LNM, lymph node metastasis. The locus analysed is provided at the bottom. The lost alleles are indicated (<)

Analysis of instability in chromosome 19 microsatellites by Weber and Wong (1993) showed a clear preference for expansions rather than contractions, with 77.5% of instabilities presenting as a larger allele. In contrast, most instabilities observed in this study were contractions, with only 3 of 13 (23%) expansion instabilities seen. The reason for this discrepancy is unknown. The observation that the rate of instability was three times greater in the tetranucleotides (2.0%) than in the dinucleotides (0.66%) has been reported previously (Weber and Wong, 1993; Mao et al, 1994). It would be worthwhile to investigate mononucleotide and pentanucleotide repeats to see whether this pattern continued. The reason for this variation in the rate of MSI between repeat types is unknown; although Weber and Wong (1993) have suggested that the discrepancy may be artefactual because of the under-reporting of instances of MSI in dinucleotides due to the masking of novel alleles by the strand slippage products common to dinucleotide repeats. If this was the case, then one would not expect to see a difference in the rate of instability between trinucleotides and tetranucleotides, assuming a common mechanism for the development of instability.

The fact that tetranucleotides appear to exhibit a greater rate of instability than dinucleotides means that the results obtained in any investigation screening for MSI will be highly dependent upon the type of repeats chosen. A study using tetranucleotides would be expected to find an incidence of MSI three times greater than another study using dinucleotides. Furthermore, there is now evidence that in particular tumour types, different microsatellite

© Cancer Research Campaign 1997

loci are inherently more likely to show instability than other loci with the same repeat length. Mao et al (1996) have described a method for detection of primary bladder cancers by microsatellite analysis. They screened a series of 60 tri- and tetranucleotide markers against 50 primary bladder cancers. They were able to rank the markers according to their susceptibility to alteration and select a panel of the 10 best microsatellite loci to use when screening for bladder cancers. Their results would suggest that, at least in bladder cancer, the rate of instability varies between microsatellites. It would be useful to determine whether the increased rates of instability seen in certain microsatellite loci are specific to a particular tumour type (tumour-specific instability) or whether these loci are generally prone to instability, regardless of the type of tumour (general tumour instability).

A problem with defining the percentage of tumours in a given study that show the $RER^+$ phenotype is that an accurate definition of what constitutes $RER^+$ does not appear to be documented. It would be of great value if all researchers could agree upon a specific panel of microsatellite markers for the screening of tumours for the presence of MSI. This would make comparisons between research groups, different tumours and different tumour types possible. Until such uniformity is achieved, direct comparisons of the results obtained by different research groups is impossible and can lead to wildly different estimates of the rate of MSI in a given tumour type (Lothe et al, 1993; Peltomaki et al, 1993; Patel et al, 1994). If the presence of any MSI is assumed to be indicative of an $RER^+$ phenotype, then the rate of $RER^+$ tumours seen in this study was 10.8%, similar to that reported by Wooster et al (1994).

There are no universal sites of genetic damage known to occur in mammary carcinoma. The background (random) rate of LOH in the mammary gland has been reported as 4% (Chen et al, 1992), and previous reports suggest a frequency of LOH at certain sites in invasive carcinoma of 19–60% (Callahan and Campbell, 1989; Ben Cheickh et al, 1992; Mooi and Peterse, 1992; Takita et al, 1992). While the D3S1514 (3p21–p14.2; Buchhagen et al, 1994), D9S254 (9p23–p21; Brenner and Aldaz, 1995) and THθ1 (11p15.5; Winqvist et al, 1993; Gudmundsson et al, 1995) loci have been reported to be important in breast carcinogenesis, the FABP and SCA3 loci were chosen as being representative of chromosomal regions thought to be irrelevant to mammary carcinogenesis. In the invasive carcinomas studied here, a similarly low rate of LOH (9.1–13%) was observed at the D3S1514, FABP, D9S254, THθ1 and SCA3 loci, which is two to three times the background rate reported by Chen et al (1992). The reason for this is unknown. The remaining three loci showed high rates of LOH (24–38%), suggesting that genes on chromosomes 17p, 17q and Xq may be important in the development of invasive mammary carcinoma.

It has been previously reported that invasive carcinomas showing LOH at 11p are more likely to metastasize (Takita et al, 1992). Our study showed that LOH at this site occurred concurrently with the development of invasive carcinoma, and an even higher rate of LOH was observed in metastases (Table 3). This suggests that LOH at this site may be associated with the acquisition of an invasive phenotype and the ability to metastasize. On chromosome 9p, LOH has previously been reported in 58% of invasive carcinomas (Brenner and Aldaz, 1995), but this study found LOH in only 11% of instances, and no instances of loss were seen in the metastatic tumours. LOH on both arms of chromosome 17 and on Xq occurred concurrently with histological features of carcinoma in situ and were present at a greater rate in invasive carcinoma. The rate of loss for all three loci was actually seen to decrease in the metastatic tumours, particularly the two chromosome 17 loci. LOH events at these sites may therefore have a role in the expression of morphological or invasive characteristics but not necessarily in the development of metastases.

There were significant associations between LOH of markers on both arms of chromosome 17 in all tissue grades (Table 5), which suggests that allelic loss at these sites may be separate but associated events. It is also possible that the entire chromosome 17 is deleted – study of additional chromosome 17 markers would confirm the loss of the entire chromosome. We studied the D17S855 microsatellite located within the *BRCA1* gene, whose mutation is associated with the development of familial mammary carcinoma occurring at an early age (Chan, 1995). Loss of heterozygosity at D17S855 suggests deletion of all or part of the gene. To date, the timings of *BRCA1* mutations and losses of heterozygosity in mammary carcinogenesis have not been studied. We observed one instance of loss at D17S855 in eleven epithelial hyperplasias, 22% loss in carcinoma in situ and a 32% rate of loss in invasive carcinomas; in metastatic carcinomas, the rate of loss decreased to 18%. Our data imply that LOH of *BRCA1* may be an event that occurs early in mammary carcinogenesis (around the stage of carcinoma in situ) and may also be observed in some epithelial hyperplasias. In patient 80, samples of epithelial hyperplasia, carcinoma in situ and invasive ductal carcinoma were analysed. Loss at the D17S855 locus was observed in the carcinoma in situ and the invasive tumour DNA but not in the epithelial hyperplasia. A high rate of LOH on 17q in invasive carcinoma and the fact that only 1–2% of mammary carcinomas carry detectable mutation in the *BRCA1* gene (Chan, 1995) implies the presence of other genes in the region that may have a role in mammary carcinogenesis. Cropp et al (1993) have identified three discrete regions of chromosome 17q that are frequently deleted in mammary carcinomas.

Despite a high frequency of LOH of D17S559 on 17p, there was no correlation with mutation in exons 5–9 of the *p53* gene (unpublished data) located on this arm ($P = 0.0683$ using Fisher's exact test), a finding supported by that of Deng et al (1994). Other workers have noted that, in 60% of breast carcinomas showing LOH at the *p53* site, the retained allele is native, i.e. not mutated (Moll et al, 1992). This implies that there may be other target sites on this chromosome arm (Sato et al, 1991).

LOH at 3p showed a statistically significant association with mutations in exons 5–9 of *p53* ($P = 0.0148$ using Fisher's exact test), which is located on chromosome 17p. This has been previously reported by Deng et al (1994). There were also significant associations between LOH on chromosomes 3p and 17p ($P = 0.0193$ for invasive carcinoma alone; $P = 0.0151$ for hyperplasia, carcinoma in situ, invasive and metastatic carcinoma). A major role of *p53* is to act as a checkpoint factor by halting cell replication unless the genome is intact. Therefore, loss of normal *p53* activity might allow persistence of errors in genes located on 3p. It is also possible that mutations in *p53* occur after LOH on 3p, particularly as chromosome 3p carries the DNA mismatch repair gene *hMLH1*. LOH at this site might result in a down-grading of the cell's ability to repair errors of replication.

When the rates of LOH seen in all cases with MSI was compared with the rates of LOH in all cases without MSI, there were no apparent differences. Of the 13 cases that showed MSI, seven (53.8%) showed no loss, while 82 of 151 (54.3%) cases without MSI also showed no evidence of LOH. In the remaining six cases with MSI, there were 13 occurrences of LOH. The number of

 © Cancer Research Campaign 1997

occurrences of loss at each locus was as follows: D3S1514 (two), FABP (one), D9S254 (zero), THθ1 (one), SCA3 (two), D17S559 (three), D17S855 (three) and HUMARA (one). The only case that showed LOH at five of the eight loci tested also showed MSI, while the single case that showed more than one instance of MSI showed no LOH. Thus, there was no obvious association between the occurrence of LOH and MSI within samples.

Overall, the occurrence of any microsatellite alteration was 33% in the epithelial hyperplasias and 32% in the carcinomas in situ, but this almost doubled to 65% in the invasive tumours. Aldaz et al (1995) reported that 74% of 23 ductal carcinomas in situ showed at least one alteration in a panel of 20 microsatellite markers. The reason for the discrepancy between their results and those obtained here may be attributed to the larger number of microsatellite markers they used, as well as the locations of those markers. The number of metastatic tumours analysed in this study that showed at least one alteration was 46%, lower than for the invasive tumours. The reason for this is unknown. The rates of LOH reported by Aldaz et al (1995) for the ductal carcinomas in situ are in close agreement with the results obtained in this study. They reported no losses at 3p or 11p and high rates (25–30%) of loss at 17p and 17q. We saw no LOH at 3p or 11p and a rate of LOH of 24% at 17p and 22% at 17q.

The findings of this investigation suggest that, unlike hereditary non-polyposis colorectal carcinoma, the RER+ phenotype is uncommon, and MSI does not play a significant role in the initiation or progression of most instances of mammary carcinomas. We did not observe a significant occurrence of MSI in the tumours examined. MSI was seen in epithelial hyperplasias, carcinomas in situ and invasive carcinomas, but the rate of instability observed did not alter. This would indicate that the MSI seen in most mammary neoplasms is some form of epiphenomenon that is of neutral effect, not providing the tumour with either a selective growth advantage or disadvantage. In contrast, LOH was a more common phenomenon than MSI in all tissues studied, indicating that it may be an important mechanism in the pathogenesis of breast cancer.

### ACKNOWLEDGEMENTS

EKD is the recipient of a Raine Medical Research Foundation scholarship. GRT is the recipient of a Raine Medical Research Foundation Grant. We are grateful to the staff of the Royal Perth Hospital Department of Pathology for preparation of the paraffin-embedded tissues and slides and to Dr Cecily Metcalf for some of the initial case selection.

### REFERENCES

Aaltonen LA, Peltomaki P, Leach FS, Sistonen P, Pylkkanen L, Mecklin J-P, Jarvinen H, Powell SM, Jen J, Hamilton SR, Petersen GM, Kinzler KW, Vogelstein V and de la Chapelle A (1993) Clues to the pathogenesis of familial colorectal cancer. *Science* **260**: 812–816

Aldaz CM, Chen T, Sahin A, Cunningham J and Bondy M (1995) Comparative allelotype of *in situ* and invasive human breast cancer: high frequency of microsatellite instability in lobular breast carcinomas. *Cancer Res* **55**: 3976–3981

Bassam BJ, Caetano-Anollés G and Gresshoff PM (1991) Fast and sensitive silver staining in polyacrylamide gels. *Anal Biochem* **196**: 80–83

Ben Cheickh M, Rouanet P, Louason G, Jeanteur P and Theillet C (1992) An attempt to define sets of cooperating genetic alterations in human breast cancer. *Int J Cancer* **51**: 542–547

Brenner AJ and Aldaz CM (1995) Chromosome 9p allelic loss and *p16/CDKN2* in breast cancer and evidence of *p16* inactivation in immortal breast epithelial cells. *Cancer Res* **55**: 2892–2895

Bronner CE, Baker SM, Morrison PT, Warren G, Smith LG, Lescoe MK, Kane M, Earabino C, Lipford J, Lindblom A, Tannergard P, Bollag RJ, Godwin AR, Ward DC, Nordenskjold M, Fishel R, Kolodner R and Liskay RM (1994) Mutation in the DNA mismatch repair gene homologue *hMLH1* is associated with hereditary non-polyposis colon cancer. *Nature* **368**: 258–261

Buchhagen DL, Qiu L and Etkind P (1994) Homozygous deletion, rearrangement and hypermethylation implicate chromosome region 3p14.3–3p21.3 in sporadic breast-cancer development. *Int J Cancer* **57**: 473–479

Callahan R and Campbell G (1989) Mutations in human breast cancer: an overview. *J Natl Cancer Inst* **81**: 1780–1786

Chan JKC (1995) Breast cancer susceptibility genes make the headlines. *Adv Anat Pathol* **2**: 129–131

Chen L, Kurisu W, Ljung B-M, Goldman ES, Moore D and Smith HS (1992) Heterogeneity for allelic loss in human breast cancer. *J Natl Cancer Inst* **84**: 506–510

Cropp CS, Champeme M-H, Lidereau R and Callahan R (1993) Identification of three regions on chromosome 17q in primary human breast carcinomas which are frequently deleted. *Cancer Res* **53**: 5617–5619

Dams E, Van de Kelft EJZ, Martin J-J, Verlooy J and Willems PJ (1995) Instability of microsatellites in human gliomas. *Cancer Res* **55**: 1547–1549

Deng G, Chen L-C, Schott DR, Thor A, Bhargava V, Ljung B-M, Chew K and Smith HS (1994) Loss of heterozygosity and *p53* gene mutation in breast cancer. *Cancer Res* **54**: 499–505

Gudmundsson J, Barkardottir RB, Eiriksdottir G, Balsursson T, Arason A, Egilsson V and Ingvarsson S (1995) Loss of heterozygosity at chromosome 11 in breast cancer: association of prognostic factors with genetic alterations. *Br J Cancer* **72**: 696–701

Han H-J, Yanagisawa A, Kato Y, Park J-G and Nakamura Y (1993) Genetic instability in pancreatic cancer and poorly differentiated type of gastric cancer. *Cancer Res* **53**: 5087–5089

Han H-J, Maruyama M, Baba S, Park J-G and Nakamura Y (1995) Genomic structure of human mismatch repair gene, *hMLH1*, and its mutation analysis in patients with hereditary non-polyposis colorectal cancer (HNPCC). *Hum Mol Genet* **4**: 237–242

Hearne CM, Ghosh S and Todd JA (1992) Microsatellites for linkage analysis of genetic traits. *Trends Genet* **8**: 288–294

Horii A, Han H-J, Shimada M, Yanagisawa A, Kaot Y, Ohta H, Yasui W, Tahara E and Nakamura Y (1994) Frequent replication errors and microsatellite loci in tumours of patients with multiple primary cancers. *Cancer Res* **54**: 3373–3375

Huang TH-M, Yeh PL-H, Martin MB, Straub RE, Gilliam TC, Caldwell CW and Skibba JL (1995) Genetic alterations of microsatellites on chromosome 18 in human breast carcinoma. *Diagn Mol Pathol* **4**: 66–72

Kwiatkowski DJ, Henske EP, Weimar K, Ozelius L, Gusella JF and Haines J (1992) Construction of a GT polymorphism map of human 9p. *Genomics* **12**: 229–240

Leach FS, Nicolaides NC, Papadopoulos N, Liu B, Jen J, Parsons R, Peltomäki P, Sistonen P, Aaltonen LA, Nyström-Lahti M, Guan X-Y, Zhang J, Meltzer PS, Yu JW, Kao FT, Chen DJ, Cerosaletti KM, Fournier REK, Todd S, Lewis T, Leach RJ, Naylor SL, Weissenbach J, Mecklin JP, Järvinen P, Petersen GM, Hamilton SR, Green J, Jass J, Watson P, Lynch HT, Trent JM, de la Chapelle A, Kinzler KW and Vogelstein B (1993) Mutations of a *mutS* homolog in hereditary nonpolyposis colorectal cancer. *Cell* **75**: 1215–1225

Levine AJ (1995) The genetic origins of neoplasia. *JAMA* **273**: 592

Liu B, Parsons RE, Hamilton SR, Petersen GM, Lynch HT, Watson P, Markowitz S, Willson JKV, Green J, de la Chapelle A, Kinzler KW and Vogelstein B (1994) *hMSH2* mutations in hereditary nonpolyposis colorectal cancer kindreds. *Cancer Res* **54**: 4590–4594

Liu B, Nicolaides NC, Markowitz S, Willson JKV, Parsons RE, Jen J, Papdopolous N, Peltomaki P, de la Chapelle A, Hamilton SR, Kinzler KW and Vogelstein B (1995) Mismatch repair gene defects in sporadic colorectal cancers with microsatellite instability. *Nature Genet* **9**: 48–55

Loeb LA (1994) Microsatellite instability: marker of a mutator phenotype in cancer. *Cancer Res* **54**: 5059–5063

Lothe RA, Peltomäki P, Meling GI, Aaltonen LA, Nyström-Lahti M, Pylkkanen L, Heimdal K, Andersen TI, Moller P, Rognum TO, Fossa SD, Haldorsen T, Langmark F, Brogger A, de la Chapelle A and Borresen A-L (1993) Genomic instability in colorectal cancer: relationship to clinicopathological variables and family history. *Cancer Res* **53**: 5849–5852

Mao L, Lee DJ, Tockman MS, Erozan YS, Askin F and Sidransky D (1994) Microsatellite alterations as clonal markers for the detection of human cancer. *Proc Natl Acad Sci USA* **91**: 9871–9875

© Cancer Research Campaign 1997

Mao L, Schoenberg MP, Scicchitano M, Erozan YS, Merlo A, Schwab D and Sidransky D (1996) Molecular detection of primary bladder cancer by microsatellite analysis. Science **271**: 659–662

Mironov NM, Aguelon MA-M, Potapova GI, Omori Y, Gorbunov OV, Klimenkov AA and Yamasaki H (1994) Alterations of $(CA)_n$ DNA repeats and tumour suppressor genes in human gastric cancer. *Cancer Res* **54**: 41–44

Moll UM, Tiou G and Levine AJ (1992) Two distinct mechanisms alter *p53* in breast cancer: mutation and nuclear exclusion. *Proc Natl Acad Sci USA* **89**: 7262–7266

Mooi WJ and Peterse JL (1992) Progress in molecular biology of breast cancer. *Eur J Cancer* **28**: 623–625

Nicolaides NC, Papadopoulos N, Liu B, Wei Y-F, Carter KC, Ruben SM, Rosen CA, Haseltine WA, Fleischmann RD, Fraser CM, Adams MD, Venter JC, Dunlop MG, Hamilton SR, Petersen GM, de la Chapelle A, Vogelstein B and Kinzler KW (1994) Mutations of two *PMS* homologues in hereditary nonpolyposis colon cancer. *Nature* **371**: 75–80

Papadopoulos N, Nicolaides NC, Wei Y-F, Ruben SM, Carter KC, Rosen CA, Haseltine WA, Fleischmann RD, Fraser CM, Adams MD, Venter JC, Hamilton SR, Petersen GM, Watson P, Lynch HT, Peltomäki P, Mecklin J-P, de la Chapelle A, Kinzler KW and Vogelstein B (1994) Mutation of a *mutL* homolog in hereditary colon cancer. *Science* **263**: 1625–1629

Parsons R, Li G-M, Longley MJ, Fang W-H, Papadopoulos N, Jen J, de la Chapelle A, Kinzler KW, Vogelstein B and Modrich P (1993) Hypermutability and mismatch repair deficiency in $RER^+$ tumour cells. *Cell* **75**: 1227–1236

Patel U, Grundfest-Broniatowski S, Gupta M and Banergee S (1994) Microsatellite instabilities at five chromosomes in primary breast tumours. *Oncogene* **9**: 3695–3700

Peltomäki P, Lothe RA, Aaltonen LA, Pylkkanen L, Nyström-Lahti M, Seruca R, David L, Holm R, Tyberg D, Haugen A, Brogger A, Borresen A-L and de la Chapelle A (1993) Microsatellite instability is associated with tumours that characterize the hereditary non-polyposis colorectal carcinoma syndrome. *Cancer Res* **53**: 5853–5855

Sato T, Akiyama F, Sakamoto G, Kasumi F and Nakamura Y (1991) Accumulation of genetic alterations and progression of primary breast cancer. *Cancer Res* **51**: 5794–5799

Takita KI, Sato T, Miyagi M, Watatani M, Akiyama F, Sakamoto G, Kasumi F, Abe R and Nakamura Y (1992) Correlation of loss of alleles on the short arms of chromosomes 11 and 17 with metastasis of primary breast cancer to lymph nodes. *Cancer Res* **52**: 3914–3917

Turbett GR, Barnett TC, Dillon EK and Sellner LN (1996) A single-tube protocol for the extraction of DNA or RNA from paraffin embedded tissue using a starch-based adhesive. *Biotechniques* **20**: 846–853

Weber JL and Wong C (1993) Mutation of human short tandem repeats. *Hum Mol Genet* **2**: 1123–1128

Willems PJ (1994) Dynamic mutations hit double figures. *Nature Genet* **8**: 213–215

Winqvist R, Mannermaa A, Alavaikko M, Blanco G, Taskinen PJ, Kiviniemi H, Newsham I and Cavenee W (1993) Refinement of regional loss of heterozygosity for chromosome 11p15.5 in human breast tumors. *Cancer Res* **53**: 4486–4488

Wooster R, Cleton-Jansen A-M, Collins N, Mangion J, Cornelis RS, Cooper CS, Gusterson BA, Ponder BAJ, von Deimling A, Wiesler OD, Cornelisse CJ, Devilee P and Stratton MR (1994) Instability of short tandem repeats (microsatellites) in human cancers. *Nature Genet* **6**: 152–156

Yee CJ, Roodi N, Verrier CS and Parl FF (1994) Microsatellite instability and loss of heterozygosity of breast cancer. *Cancer Res* **54**: 1641–1644

© Cancer Research Campaign 1997